# Structured implementation of a bubble-CPAP program to reduce bronchopulmonary dysplasia in preterm infants

**DOI:** 10.1038/s41390-025-04635-4

**Published:** 2025-12-09

**Authors:** Hany Aly, Hany Aziz, Vanishree Nandakumar, Firas Saker, Mohamed A. Mohamed

**Affiliations:** Cleveland Clinic Children’s Hospital, Cleveland, OH USA

## Abstract

**Background:**

CPAP effectiveness may depend not only on the device itself, but also on implementation. We hypothesized that structured implementation would be associated with improved outcomes. This study describes a bubble-CPAP (b-CPAP) program—including training, guidelines, skills practice, and checklists—and evaluates its clinical correlates.

**Methods:**

This study describes b-CPAP program implementation. The study included all preterm infants with birthweight <1500 g. Infants were excluded if gestational age <22 weeks, died in the delivery room before NICU admission, had genetic conditions or surgical conditions requiring transfer. Data were collected from January 2016 through December 2022. Outcomes before and after implementation were compared using bivariate and logistic regression analyses while adjusting for potential confounders.

**Results:**

Among 903 infants, survival without BPD improved from 59% to 74% (aOR=1.86, 95% CI: 1.25–2.76, *p* = 0.01), and BPD rates decreased from 25.2% to 6.9% (aOR = 0.12, 95% CI:0.06–0.26, *p* < 0.001). Median oxygen use decreased from 26 (4–70) days to 15 (4–42) days (*p* = 0.03), and CPAP use increased from 10 (5–19) days to 27 (10–39) days (*p* < 0.0001). The incidence of pneumothorax did not change (4.1% to 6.4%, aOR = 1.59, 95% CI:0.80–3.18, *p* = 0.18).

**Conclusion:**

Stepwise implementation of a structured b-CPAP program was associated with substantial improvements in survival without BPD and BPD rates.

**Impact:**

- Bronchopulmonary dysplasia (BPD) remains a major complication in premature infants, affecting approximately 40% of infants born under 1000 grams.
- While RCTs showed only modest benefit of early CPAP, observational studies reported greater improvements, underscoring the importance of consistent implementation beyond initial use.
- Structured stepwise implementation of the b-CPAP program was associated with improved survival without BPD and reduced BPD prevalence.
- The findings may inform future strategies to enhance reproducibility and effectiveness of CPAP use through systematic implementation.

## Introduction

Bronchopulmonary dysplasia (BPD) remains a major complication in premature infants. Its prevalence increases with decreasing gestational age (GA) and birth weight (BW), affecting approximately 40% of infants born under 1000 grams.^1^ With improving survival among extremely preterm infants, the burden of BPD continues to grow, carrying long-term risks of mortality and neurodevelopmental impairment.^1^ While lifesaving, mechanical ventilation and supplemental oxygen are also major contributors to lung injury, and thus represent modifiable targets in BPD prevention strategies.^2^

A Cochrane meta-analysis demonstrated that early use of continuous positive airway pressure (CPAP) compared with routine intubation for prophylactic surfactant is associated with improved survival without BPD (RR 0.90, 95% CI: 0.83–0.98 risk difference −0.04 (95% confidence interval −0.08 to −0.00), NNT of 25).^3^ Observational studies have reported an even greater reduction in BPD rates with early CPAP use.^4,5^ Although such studies are subject to bias and confounding, the direction of effect has been consistent with the trial evidence. Notably, these observational reports often incorporated practices extending beyond initial stabilization, including prolonged use of CPAP, avoidance of high-flow nasal cannula or narrow-bore interfaces, and application of bubble CPAP techniques. The observed variation in effect size between trial data and real-world experience underscores that implementation quality likely modulates CPAP efficacy. These findings raise a critical question: are favorable CPAP outcomes attributable to the device, or to the way it is implemented? Many NICUs, including ours, have long used CPAP—yet without consistently achieving the outcomes reported by expert centers. We therefore hypothesized that successful CPAP use depends not solely on the device, but on a structured implementation approach. In response, we developed a ‘bubble-CPAP (b-CPAP) program’ encompassing hands-on training, bedside skills practice, protocolized guidelines, and competency-based checklists.^4^ This program aims to standardize care, promote caregiver proficiency, and create a shared mental model for CPAP use.

This study aimed to describe the implementation of a structured bubble-CPAP (b-CPAP) program in 2 NICUs already utilizing CPAP —incorporating hands-on training, standardized guidelines, bedside skills practice, and competency checklists — and to evaluate its associated clinical outcomes. We hypothesized that introducing the b-CPAP program would be associated with improved survival without BPD and a reduced incidence of BPD in preterm infants with BW < 1500 g. The findings may inform future strategies and clinical trials to enhance reproducibility and effectiveness of CPAP use through systematic implementation.

## Methods

### Patients

The implementation study included all infants with BW < 1500 g who were admitted to the NICUs between January 1^st^, 2016, and December 31^st^, 2022. Infants were excluded from the study if they had any of the following conditions: (a) GA < 22 weeks or >34 weeks, (b) genetic syndromes or major congenital anomalies, (c) death in the delivery room before NICU admission, and (d) surgical conditions requiring transfer out of the NICU before 36 weeks of postmenstrual age (PMA). Infants who died or were transferred at 36 weeks or greater were included in the analysis. The institutional review board at the Cleveland Clinic approved this study before any data collection. Parental consent was not required, as all infants were managed according to the new b-CPAP guidelines in each hospital.

### Setting

This implementation study was conducted in the greater Cleveland area, where Cleveland Clinic operates two Level III NICUs, managing ~10,000 deliveries and 1300 NICU admissions annually. Care teams included physicians, advanced neonatal practitioners, and respiratory therapists, with dedicated nursing staff at each unit. Before the introduction of the program, infants with respiratory distress were intubated in the delivery room (DR) using a less restrictive threshold, mechanically ventilated, and given surfactant via endotracheal tube. There were no standardized guidelines for extubation, post-extubation respiratory support, or reintubation criteria. The methods for non-invasive respiratory support were not standardized, including the use of high flow nasal cannula, different CPAP apparatuses, and biphasic CPAP. Facial interfaces were not standardized, including nasal prongs, facial masks, and RAM cannula, without formal training or reliability measures to ensure consistent application.^6^

### B-CPAP guidelines

B-CPAP device description and guidelines were previously published, and details are provided in Supplement File A.^4,7^ Table 1 summarizes the fundamental differences between the newly adopted b-CPAP guidelines and the former CPAP practice in the enterprise. An essential component of the guidelines starts in the DR where infants are assessed, and those with spontaneous respiratory efforts are supported with CPAP at 5 cm H₂O using a facial mask attached to an anesthesia bag or to a T-piece connector (Neopuff, Fisher & Paykel, Auckland, New Zealand). Infants are then immediately transported to the NICU, where a preset b-CPAP unit is ready at the bedside. Short curved nasal prongs, such as Hudson RCI nasal prongs (Teleflex, Auckland, New Zealand) or Babi.Plus nasal prongs (Respiralogics, Reno, NV), are used as the facial interface, and the b-CPAP pressure is set to 5 cm H₂O, with the option to increase pressure to 6 cm H₂O to ensure comfortable spontaneous breathing efforts.

**Table 1:** Components of CPAP practice before and after the implementation of the new guidelines.

Element	Baseline CPAP practice	New b-CPAP guidelines
Delivery room management	Preterm infants are intubated, and surfactant is administered shortly after	CPAP is the primary mode of support. Preterm infants are intubated if they have apnea or if unable to achieve goal saturation while supported with CPAP
CPAP device	Machine generated via ventilators, infant flow apparatus, or biphasic CPAP device	Only bubble CPAP
Facial interface	Facial mask, binasal prongs, or RAM cannula	Only short curved binasal prongs
CPAP pressure	No limits; ranging from 4 to 12 cmH_2_O	5 cmH_2_O with the option to increase to 6 cmH_2_O if no improvement
CPAP failure criteria	Unspecified	FiO2 > 50% in the first day or >60% thereafter, clinically significant apnea, and PCO_2_ > 65 mmHg accompanied with metabolic acidosis (BD > 10 mmol/L)
CPAP weaning criteria	Unspecified- frequent transition HFNC	Weaning of FiO_2_ until reaches 21%. No use of HFNC. No pressure wean. CPAP is tried off once a day until successful.
CPAP exchange with nasal cannula or HFNC	Yes	Not permitted
Respiratory support after extubation	NIPPV, CPAP, HFNC	Bubble CPAP
CPAP implementation tool		
Bedside CPAP training	No	Yes
Bedside checklist	No	Yes
CPAP guidelines	No	Yes
Reliability measure	No	Yes

*b-CPAP* bubble CPAP, *BD* base deficit, *CPAP* continuous positive airway pressure, *DR* delivery room, *FiO*_2_ fraction of inspired oxygen, *HFNC* high flow nasal cannula, *NICU* neonatal intensive care nursery, *NIPPV* non-invasive positive pressure ventilation, *PCO*_2_ partial pressure of carbon dioxide.

If the fraction of inspired oxygen cannot be weaned to less than 50% within the first four hours of b-CPAP, the infant is intubated, and surfactant replacement therapy is administered. In cases where the infant does not breathe spontaneously in the DR, despite stimulation and positive pressure ventilation via bag-and-mask, intubation is performed, and conventional mechanical ventilation is initiated. Surfactant replacement therapy is administered if supplemental oxygen is required, and the infant shows radiological signs of respiratory distress syndrome.

### CPAP failure criteria

Infants are intubated if the required oxygen fraction is ≥ 0.50 on the first day of life or ≥60% thereafter while being supported with b-CPAP at a pressure of 5–6 cm H₂O. The current NICU guidelines do not support increased CPAP pressures >6 cm H₂O, as the nasal prongs used in these guidelines are short, wide, and snugly fit into the nostrils, which is believed to deliver effectively the intended pressure to the lower airways. Premature infants are not intubated solely based on perceived work of breathing or chest retractions, as these clinical signs may often be attributed to the high compliance of the chest wall — which is typical in premature infants — rather than to respiratory insufficiency. Infants experiencing frequent apnea and bradycardia are managed initially with non-invasive mechanical ventilation before considering intubation.

### Weaning criteria

Oxygen concentration is adjusted to maintain transcutaneous oxygen saturation between 90% and 95%. Once oxygen can be weaned to 21% for a full 24-hour period, infants are allowed to attempt weaning off b-CPAP no more than once per day. If the infant does not tolerate the wean, b-CPAP is resumed. Nasal cannulas are not used at any stage during the b-CPAP weaning process. Infants born at the lowest gestational ages (22–25 weeks) and with a BW < 1000 g are not weaned off b-CPAP until their weight reaches 1300 g.

### B-CPAP implementation

In October 2017, an implementation trial of the b-CPAP program was initiated in one of the two hospitals (Hospital A). Leadership approval was secured following discussions and a literature review on potential benefits and challenges. A NICU steering committee, composed of physicians, advanced practitioners, nurses, and respiratory therapists, was formed to manage the change process. Members attended a b-CPAP workshop and visited a NICU with extensive experience in b-CPAP use to gain practical insights.^4^

By January 2018, b-CPAP guidelines (see Supplementary file) were developed, and necessary supplies were procured for Hospital A. Training involved interactive lectures on standardized b-CPAP use, hands-on simulation-based practice, and a competency checklist (Supplementary File B). On February 1, 2018, b-CPAP was introduced for infants >1500 g requiring respiratory support. Biweekly meetings reviewed usage data, complications, and staff feedback, with progress reports displayed on the NICU bulletin board. As proficiency increased, eligibility expanded to infants >1250 g, and by December 2018, b-CPAP was used for all preterm infants, regardless of BW or GA.

Process reliability was ensured through bedside audits by nurse champions, who provided real-time feedback on headcap placement, breathing tube positioning, nasal prongs fit, and checklist completion. Monthly compliance data were shared with staff to promote adherence and engagement.

On January 1, 2019, b-CPAP implementation expanded to Hospital B, mirroring the process at Hospital A. Full implementation was achieved by December 2019, with occasional support from experienced nurses and respiratory therapists from Hospital A, facilitating a smoother transition.

This phased approach ensured systematic adoption, improved provider proficiency, and established a sustainable b-CPAP program with high reliability and engagement across both NICUs.

### Other management

No intended changes in clinical practice were made during the study period, aside from the introduction of the b-CPAP program.

### Data collection

Maternal history was obtained, including maternal age, gravidity, and pregnancy complications such as hypertension, diabetes, and infections. Data collected from the infants included GA, BW, sex, Apgar scores at 1 and 5 min, intubation in the delivery room, sepsis, pulmonary hemorrhage, necrotizing enterocolitis, retinopathy of prematurity, and bronchopulmonary dysplasia (BPD). Data were prospectively collected by an investigator who was not involved in the analysis. Data were collected on process measures, including the duration of CPAP, mechanical ventilation, and supplemental oxygen use. Reliability checks were performed to assess compliance with established guidelines, and balancing measures such as nasal injury and pneumothorax were also recorded. Baseline data for the years 2016–2017 were collected retrospectively.

### Power analysis

The benchmark for survival without BPD in VLBW infants was previously reported at 61.1%.^8^ To detect 20% relative increase up to 73.3%, a sample of 229 infants would be required for analysis (*α* = 0.05 and *β* = 0.2). Considering an attrition rate of 15% related to infants’ transfer for surgical interventions and/or other missing data, a sample of 263 infants in the pre- and 263 infants in the post-implementation population would suffice.

### Analytic plan

The analytic plan was established before the start of the study. Data were categorized into three time frames: Period 1 (January 1^st^, 2016 – December 31^st^, 2017) consisted of baseline data from both units obtained before the b-CPAP implementation trial; Period 2 (January 1^st^, 2018 – December 31^st^, 2019) represented the combined duration of the b-CPAP introduction to both units; and Period 3 (January 1^st^, 2020 – December 31^st^, 2022) represented the time after the full establishment of the b-CPAP program in both units. The primary outcome of this study was the rate of survival without BPD at 36 (0/7) weeks of PMA, compared across the entire cohort before and after the implementation of the b-CPAP program (Period 1 vs. Period 3).

Maternal demographics and clinical variables, as well as infants’ demographic and clinical outcomes, were analyzed using normal distribution methods. Continuous variables were represented using means and standard deviations or medians and interquartile ranges, while categorical variables were represented using frequencies and percentages. The prevalence of mortality, transport out of the hospital, BPD, and the rate of survival were calculated using the Mantel-Haenszel, Chi-square, and Fisher exact tests. Logistic regression analyses were conducted to compare outcomes while controlling for confounding variables, including the use of prenatal steroids and magnesium sulfate, chorioamnionitis, GA, BW, race/ethnicity, sex, and the need for intubation in the delivery room.

Statistical significance was determined when *p* < 0.05. All analyses were performed based on the intention to treat by one of the investigators (MAM) using SAS version 9.4 (Cary, NC).

### Outcome measures

The primary outcome measure was the rate of survival without BPD at 36 0/7 weeks of PMA; this definition represents the standard for BPD at the start of the project. To maintain consistency with previous randomized controlled trials on the efficacy of CPAP in premature infants, this study adopted the same definition used in those trials: the use of oxygen at 36 0/7 weeks of post-conceptual age. New definitions for BPD were not available at the time of CPAP implementation in 2018. Secondary outcomes included the incidence of BPD and mortality at ≤36 weeks of PMA. Non-pulmonary outcomes included severe intraventricular hemorrhage, severe retinopathy of prematurity, and necrotizing enterocolitis.

#### Subgroup analyses

Subgroup analyses were performed pre- and post-implementation of the b-CPAP program, stratifying the population into three GA categories: 22–25 weeks, 26–29 weeks, and 30–34 weeks. Infants born on any day of the gestational week were analyzed according to the specific week (e.g., infants with a GA of 24 weeks and 6 days were categorized as 24-week infants).

Comparisons were made between Hospital A and Hospital B during the implementation period (2018–2019), enabling the concurrent assessment of the primary outcome in Hospital A, where the b-CPAP program had already been established, and in Hospital B, where the program had not yet been implemented.

#### StaRI compliance statement

This study reports the implementation of the b-CPAP program in accordance with the Standards for Reporting Implementation Studies (StaRI) guidelines.^9^ Consistent with StaRI, we distinguish between the clinical intervention (bubble CPAP as the primary respiratory support) and the implementation strategy (structured program comprising training, bedside checklists, audit and feedback, and phased adoption). Both the implementation processes and the associated clinical outcomes are described.

## Results

**Fig. 1:**
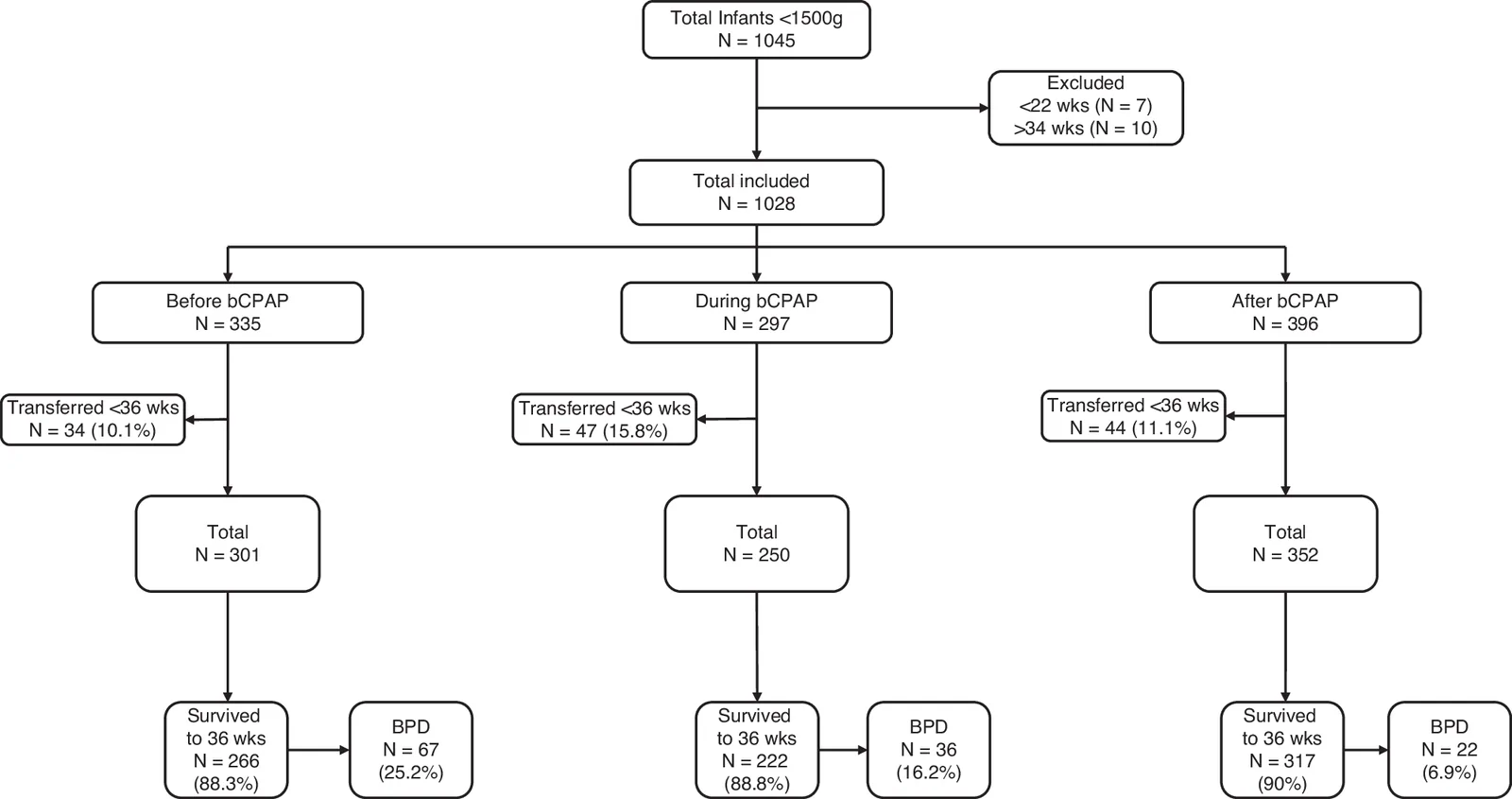
Distribution of the study population. This figure illustrates the distribution of infants included in the study across the three designated time frames: before, during and after b-CPAP implementation.

A total of 1045 infants with BW < 1500 g were considered for this study between January 1^st^, 2016, and December 31^st^, 2022. Seven infants were born at <22 weeks of gestation, and 10 infants were >34 weeks of gestation; they were excluded from the analysis. A flowchart of participants is presented in Fig. 1. The disposition of infants by GA is detailed in the supplementary file (Table S1).

Table 2 demonstrates the maternal, perinatal, and infant demographics of the overall population during the pre- and post-implementation periods. There was no difference between groups in maternal demographics or mode of delivery. The median GA (28 vs. 28 weeks, *p* = 0.93) and mean BW (1026 vs. 1027 g, *p* = 0.97) did not differ between the two groups. The frequency of delivery room intubation decreased significantly from pre- to post-implementation (48.7% vs 33.8%, OR = 0.53, 95% CI: 0.40–0.73, *p* < 0.001). Surfactant utilization also decreased (60.1% vs 50.1%, OR = 0.67, 95% CI: 0.49–0.89, *p* = 0.009). Apnea of prematurity was diagnosed more frequently in the post-implementation period (89.6% vs 94.1%, *p* = 0.03). A non-significant trend for use of postnatal glucocorticoids was noticed during the two study periods (12.6% vs 17.9% vs., *p* = 0.051). No other changes were detected in the two periods.

**Table 2:** Characteristics of the study population.

	Before Intervention (*n* = 335)	After Intervention (*n* = 396)	Odds ratios (95%CI), *p*-values
**Maternal characteristics**			
Maternal age, mean (SD), years	30 (6)	29 (6)	0.12
Diabetes mellitus (%)	9.41	10.1	1.08 (0.66–1.76), 0.80
Hypertension/preeclampsia (%)	31.7	36.0	1.21 (0.89–1.65), 0.21
Chorioamnionitis (%)	11.7	10.6	0.89 (0.57–1.42), 0.64
Use of prenatal steroids (%)	90.5	92.4	1.28 (0.76–2.16), 0.36
Use of magnesium sulfate (%)	83.5	85.7	1.18 (0.79–1.77), 0.47
Placental abruption (%)	9.73	9.50	0.97 (0.59–1.59), 1.00
Cord prolapse (%)	1.78	0.50	0.28 (0.06–1.39), 0.15
**Delivery room management**			
Cesarean delivery (%)	66.9	70.3	1.17 (0.86–1.60), 0.34
Delivery room intubation (%)	48.7	33.8	0.53 (0.40–0.73), <0.001
Apgar score at 1 min, median (IQR)	5 (2–7)	5 (2–7)	0.22
Apgar score at 5 min, median (IQR)	7 (5–8)	7 (5–8)	0.40
**Infant’s demographics**			
Gestational age, median (IQR), week	28 (25–31)	28 (26–30)	0.93
Birth weight, mean (SD), gram	1026 (332)	1027 (332)	0.97
Female sex (%)	50.9	48.4	0.91 (0.68–1.21), 0.51
Race/Ethnicity –Caucasian (%)	52.9	51.1	0.83
African American (%)	33.0	33	
Other (%)	14.1	15.9	
Singleton pregnancy (%)	73.7	77.7	1.24 (0.89–1.74), 0.23
Small for gestational age (%)	21.2	24.6	1.21 (0.85–1.74), 0.32
**Infants’ characteristics and interventions**			
Respiratory distress syndrome (%)	94.3	96.9	1.94 (0.93–4.06), 0.09
Surfactant therapy (%)	60.1	50.1	0.67 (0.49–0.89), 0.009
Apnea of prematurity (%)	89.6	94.1	1.85 (1.06–3.22), 0.03
Caffeine therapy (%)	85.8	87.5	1.15 (0.75–1.78), 0.58
Anemia of prematurity (%)	93.7	93.9	1.02 (0.55–1.89), 1.00
Thrombocytopenia (%)	46.5	46.8	1.01 (0.75–1.36), 1.00
Platelets transfusions (%)	10.4	15.4	1.56 (0.99–2.46), 0.06
Postnatal glucocorticoid use (%)	12.6	17.9	1.52 (1.00-2.29), 0.051
Discharge weight, mean (SD), g	2590 (792)	2691 (860)	0.15
Discharge head circumference, mean (SD), cm	33 (2)	33 (2)	0.23
Discharge length, mean (SD), cm	46 (4)	46 (4)	0.37
**Complications**			
Severe intraventricular hemorrhage (%)	6.03	8.85	1.51 (0.85–2.71), 0.19
Periventricular leukomalacia (%)	5.08	7.81	1.58 (0.85–2.96), 0.17
Severe retinopathy of prematurity (%)	4.68	6.20	1.35 (0.71–2.57), 0.42
Pneumothorax (%)	4.09	6.38	1.59 (0.80–3.18), 0.18
Patent ductus arteriosus (%)	32.9	27.3	0.77 (0.55–1.06), 0.11
PDA treatment (%)	14.2	9.42	0.63 (0.39–1.01), 0.06
Necrotizing enterocolitis (%)	6.99	6.07	1.16 (0.63–2.14), 0.65
Sepsis (%)	11.6	8.80	0.74 (0.45–1.21), 0.25

Adjusted OR and *p* values for binomial adverse outcome during hospitalization are calculated using logistic regression models controlling for sex, race, singleton and small for gestational age, gestational age, birth weight, maternal hypertension, diabetes mellitus, chorioamnionitis, betamethasone, or magnesium sulfate, delivery room intubation, use of surfactant therapy, caffeine, & dexamethasone. P values were calculated for continuous variables using student *t*-test while Kruskal-Wallis test was used to calculate *p* values for non-parametric continuous variables.

Survival without BPD, mortality, and BPD rates in the pre- and post-CPAP implementation periods are presented in Table 3. In the overall population, survival without BPD improved from 59.4% to 74.2% (aOR = 1.86, 95% CI: 1.25–2.76, *p* = 0.01). Mortality did not change (10% vs 9%, *p* = 0.24). The incidence of BPD decreased from 25.2% to 6.9% (aOR = 0.12, 95% CI: 0.06–0.26, *p* < 0.001). The pre- and post-implementation groups did not differ in the proportion of infants needed to transfer (10% vs 11%), respectively (OR = 1.52, CI: 0.86–2.69, *p* = 0.15). The use of non-invasive respiratory support without O_2_ at 36 weeks PCA did not differ in the pre- and post- implementation periods (10.9% vs 7.6%), respectively (OR = 1.49, CI: 0.85–2.63, *p* = 0.21).

**Table 3:** Respiratory support and associated outcomes before and after intervention.

	Before Intervention	After Intervention	Odds ratios (95%CI), *p*-values
Overall sample	*n* = 335	*n* = 396	
Respiratory support			
CPAP days^a^	10 (5–19)	27 (10–39)	*p* < 0.0001
Oxygen days^a^	26 (4–70)	15 (4–42)	*p* = 0.03
Mechanical ventilation days^a^	1 (0–5)	0 (0–3)	<0.001
Non-invasive ventilation days^a^	0 (0–7)	0 (0–7)	*p* = 0.53
Outcomes			
Survival without BPD	199 (59.4)	294 (74.2)	1.86 (1.25–2.76), 0.01
Mortality < 36–week PMA	35 (10)	35 (9)	1.47 (0.77–2.80), 0.24
BPD	67 (25.2)	22 (6.9)	0.12 (0.06–0.26), <0.001
**Infants 22–25 weeks**	***n*** = **92**	***n*** = **89**	
Respiratory support			
CPAP days^a^	18 (11–30)	43 (33–50)	*p* < 0.0001
Oxygen days^a^	100 (77–120)	76 (54–102)	*p* = 0.02
Mechanical ventilation days^a^	33 (18–52)	25 (11–36)	0.02
Non-invasive ventilation days^a^	28 (15–41)	16 (6–25)	*p* = 0.007
Outcomes			
Survival without BPD	13 (14)	41 (46)	6.44 (2.59–16.0), <0.001
Mortality ≤ 36–week PMA	29 (32)	21 (24)	0.52 (0.16–1.71), 0.96
BPD	37 (74)	10 (19)	0.02 (0.01–0.11), <0.001
**Infants 26–29 weeks**	***n*** = **125**	***n*** = **170**	
Respiratory support			
CPAP days^a^	9 (4–16)	28 (14–38)	*p* < 0.0001
Oxygen days^a^	19 (4–39)	14 (4–29)	*p* = 0.17
Mechanical ventilation days^a^	1 (1–3)	0 (0–2)	0.001
Non-invasive ventilation days^a^	0 (0–5)	0 (0–3)	*p* = 0.1
Outcomes			
Survival without BPD	84 (67)	128 (75)	1.39 (0.71–2.73), 0.34
Mortality ≤ 36–week PMA	5 (4)	14 (8)	3.19 (0.86–11.8), 0.08
BPD	24 (22)	11 (8)	0.17 (0.05–0.53), 0.002
**Infants 30–34 weeks**	***n*** = **118**	***n*** = **137**	
Respiratory support			
CPAP days^a^	5 (2–10)	8 (4–12)	*p* = 0.26
Oxygen days^a^	2 (1–7)	3 (2–6)	*p* = 0.76
Mechanical ventilation days^a^	0 (0–0)	0 (0–0)	0.004
Non-invasive ventilation days^a^	0 (0–0)	0 (0–0)	*p* = 0.5
Outcomes			
Survival without BPD	102 (86)	125 (91)	1.26 (0.49–3.22), 0.63
Mortality ≤ 36–week PMA	1 (1)	0 (0)	n/a
BPD	6 (5)	1 (1)	0.18 (0.02–1.83), 0.15

Data are presented in % except with “a” data are presented in median (IQR). *PMA* postmenstrual age, *BPD* bronchopulmonary dysplasia, *CPAP* continuous positive airway pressure.

Adjusted odds ratios (OR) and *p*-values for binomial outcome were calculated using logistic regression models controlling for sex, race, singleton and small for gestational age status, gestational age, birth weight, maternal conditions such as maternal hypertension, diabetes mellitus, perinatal conditions such as maternal chorioamnionitis, use of betamethasone, or magnesium sulfate, postnatal conditions such as delivery room intubation, use of surfactant therapy, caffeine, dexamethasone. *P*-values were calculated for continuous variables using Kruskal-Wallis Test.

CPAP use increased significantly from 10 (5–19) days to 27 (10–39) days, *p* < 0.0001, whereas oxygen use decreased from 26 (4–70) days to 15 (4–42) days (*p* = 0.03). The duration of respiratory support in the pre- and post-intervention in the overall population and the three GA categories are presented in Table 3.

Guideline adherence improved over time, with reliability rates of 71% (2019), 73% (2020), 93.8% (2021), and 94.4% (2022). This was associated with a decline in nasal pressure–related skin breakdown from 0.24, 0.10, 0.10, and 0.02 per 100 CPAP days across successive years. Pneumothorax incidence was similar in the pre- and post-implementation periods (4.1% vs. 6.4%; aOR 1.59, 95% CI 0.80–3.18; *p* = 0.18; Table 2).

In infants with a GA of 22–25 weeks, survival without BPD increased from 14% to 46% (aOR = 6.44, 95% CI: 2.59–16.0, *p* < 0.001), and BPD decreased from 74% to 19% (aOR = 0.02, 95% CI: 0.01–0.11, *p* < 0.001). In older infants with a GA of 26–29 weeks, there was no change in survival without BPD (67% vs 75%, aOR = 1.39, 95% CI: 0.71–2.73, *p* = 0.34), although BPD improved significantly from 22% to 8% (aOR = 0.17, 95% CI: 0.05–0.53, *p* = 0.002). In the 30–34 weeks population, there was no change in survival without BPD (86% vs 91%, aOR = 1.26, 95% CI: 0.49–3.22, *p* = 0.63) or in BPD rates (5% vs 1%, aOR = 0.18, 95% CI: 0.02–1.83, *p* = 0.15).

**Fig. 2:**
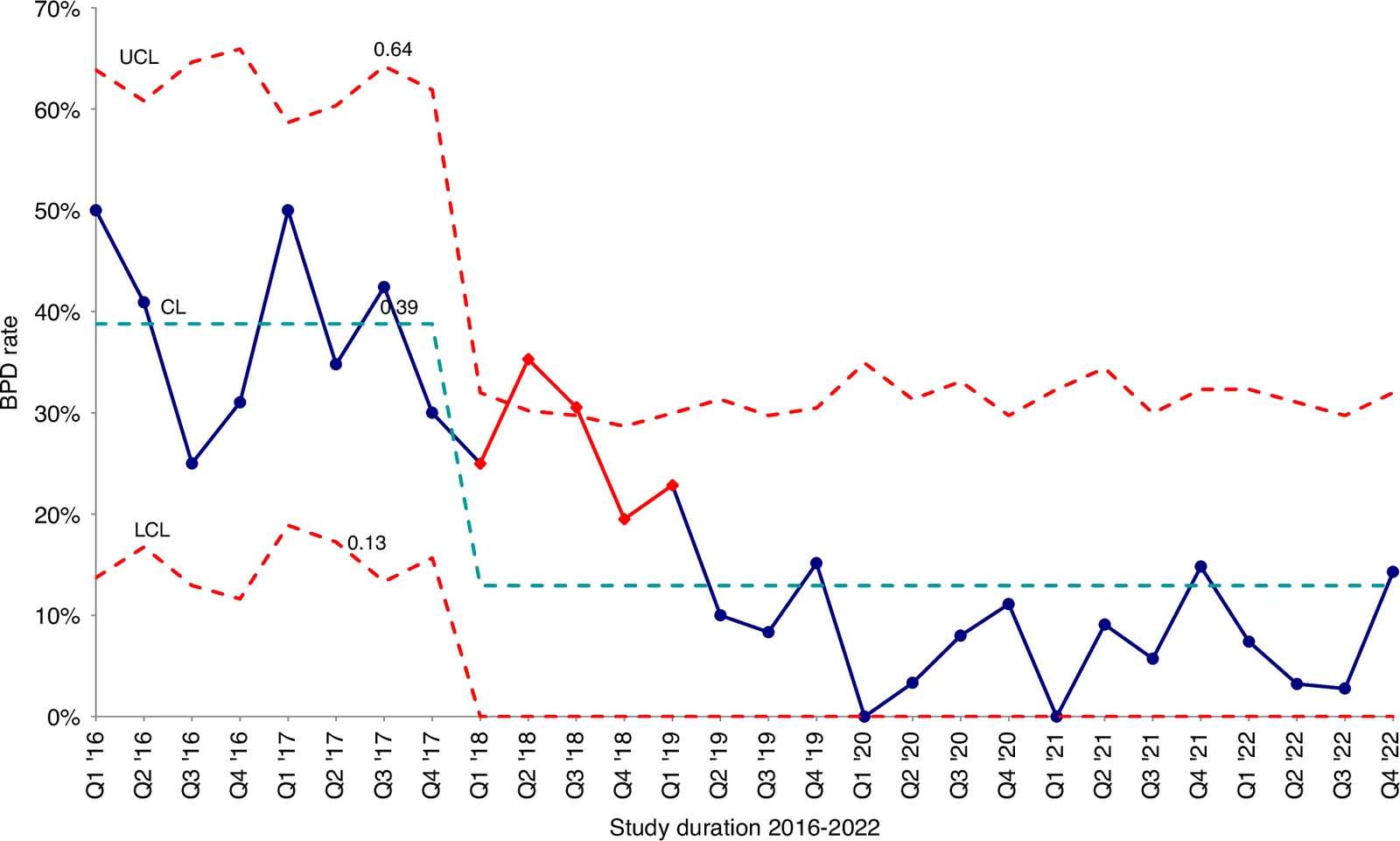
Control chart for BPD rates to evaluate the efficacy of the b-CPAP implementation program. The X-axis represents study years (2016–2022), scaled quarterly, and the Y-axis represents BPD rates among included infants. The green dashed line indicates the center line (CL), while the red dotted lines indicate the upper (UCL) and lower (LCL) control limits. By Q2 2019, five consecutive points fell below the center line, indicating a favorable special cause variation consistent with improved efficacy of the b-CPAP program.

**Fig. 3:**
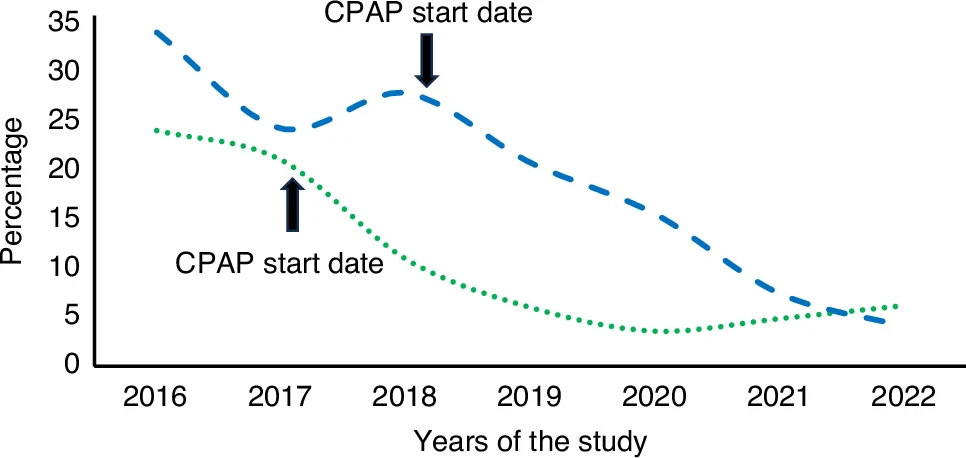
Bronchopulmonary dysplasia in the two hospitals during the study duration. The dotted green line represents Hospital A and the dashed blue line represents Hospital B. Arrows denote the initiation of the b-CPAP intervention in each hospital. Statistically significant differences in BPD prevalence in the pre- vs post-implementation periods and between the two NICUs only during the implementation phase (2018–2019).

Figure 2 presents a proportion control chart (P-Chart) of BPD prevalence, showing a favorable special cause variation in 2019. A significant decrease in BPD was noticed in each hospital. The incidence of BPD decreased from 28% to 9% (aOR = 0.17, 95% CI: 0.06–0.47, *p* < 0.001) in Hospital A, and from 23% to 5% (aOR = 0.09, 95% CI: 0.02–0.31, *p* < 0.001) in Hospital B (Fig. 3).

The two hospitals did not differ in the prevalence of BPD in the years prior to b-CPAP implementation (aOR = 0.60, CI: 0.28–1.36, *p* = 0.31 for 2016, and aOR = 0.85, CI: 0.39–1.85, *p* = 0.69 for 2017). There were also no differences between the two NICUs in the post-b-CPAP implementation years: aOR = 0.21, CI: 0.04–1.09, *p* = 0.07; aOR = 0.65, CI: 0.11–3.73, *p* = 0.69; and aOR = 1.01, CI: 0.22–4.75, *p* = 0.98 in 2020, 2021, and 2022, respectively. Hospital A had significantly lower rates of BPD in 2018 (aOR = 0.32, CI: 0.12–0.92, *p* = 0.05) and 2019 (aOR = 0.24, CI: 0.06–0.90, *p* = 0.02) (Fig. 2).

## Discussion

### Main findings

Compliance with the b-CPAP program implementation improved from 71% at initiation to 94.4% in 2022. The duration of CPAP use more than doubled with implementation of the new guidelines, while oxygen days and mechanical ventilation days decreased significantly. These practice changes were associated with a significant improvement in BPD and survival without BPD in preterm infants with BW < 1500 g, particularly in the subgroup with GA of 22–25 weeks. BPD did not differ between the two hospitals in the pre- and post-implementation periods; however, Hospital A exhibited significantly lower BPD rates than Hospital B during the implementation phase.

Compared to previous trials on CPAP efficacy, the current study possesses unique features that may explain its success. First, this study aimed to minimize variability in b-CPAP practice. All infants received b-CPAP, which specified the equipment, set pressures, facial interface, fixation methods, weaning criteria, and failure criteria. Second, conceptual education and competency-based skill training were provided prior to the implementation trial. Third, a checklist with over 20 items was utilized at the bedside to ensure uniformity of practice. Fourth, b-CPAP was gradually introduced, starting with more stable and larger infants before extending to the most premature infants. Finally, the implementation trial incorporated reliability measures to audit and self-correct practices throughout the study. These measures were not considered in previous trials, making it challenging to attribute the lack of CPAP efficacy in those studies to either ineffective devices or improper implementation. Of note, Avery et al. (1987) showed wide variation in respiratory outcomes among institutions. Columbia University, where b-CPAP was consistently used, reported the lowest incidence of BPD — underscoring the role of practice and implementation.^5^ Likewise, while b-CPAP reduced CPAP failure compared with other CPAP modes, a recent meta-analysis found no difference in mortality or BPD, highlighting that implementation may be more critical than the device itself.^10^ A recent study showed implementation of a comprehensive b-CPAP protocol in very preterm infants to associate with a significant reduction in death and severe BPD, alongside improvements in respiratory practices.^11^ The gradual rollout of the b-CPAP program allowed caregivers to acquire essential skills while managing larger, more stable infants, enabling them to become competent before applying the program to more vulnerable, extremely premature infants. This phased approach ensured systematic adoption, improved provider proficiency, and established a sustainable b-CPAP program with high reliability and engagement across both NICUs. The steering committee expanded b-CPAP use to smaller infants only after assessing caregivers’ bedside experience and monitoring their competence using the checklist. This study reports on the efficacy of b-CPAP following two years of implementation, suggesting that rapid improvements in outcomes may be unrealistic. Thus, we advocate for future trials to include a transitional phase focused on monitoring competencies and compliance with guidelines before shifting to the post-implementation phase, which emphasizes measuring clinical outcomes.

Survival without BPD improved by 25% in the overall population, and by more three folds in the 22–25 weeks GA group. The key factor underlying this success was consistency—in training, equipment, and clinical application. Inconsistent practices may explain why earlier trials showed limited benefit compared with results from experienced centers.^3–6,12^ While major trials comparing b-CPAP to other forms of CPAP or different facial interfaces are lacking, consistency in care across units enhances staff competence. It is also plausible that b-CPAP may be more efficacious than other CPAP modalities due to the oscillatory nature of the pressure provided through the bubbler.^13^ Nonetheless, as multiple elements of the b-CPAP program were implemented simultaneously, it is not possible to determine which specific component contributed most to the observed improvements; however, each element, such as initiation, duration, weaning, and failure criteria, interface selection, deimplementation of high-flow therapy, and reduction of oxygen exposure, has supporting evidence, including randomized trials, that underpins their inclusion.

The current study did not endorse the use of nasal cannula at any stage of b-CPAP implementation. Previous trials demonstrated superior efficacy for CPAP when compared to nasal cannula for respiratory support in premature infants.^14^ Weaning off invasive mechanical ventilation to CPAP is known to be more successful than weaning to nasal cannula, thereby alleviating the associated ventilator-induced lung injury. Thus, the early use of b-CPAP may effectively mitigate lung injury. CPAP has been shown to enhance lung growth over time,^15,16^ leading to a shorter duration of respiratory support and reduced oxygen needs.^14^ There is no evidence that nasal cannula use promotes lung growth; hence, infants in this implementation study were not transitioned from b-CPAP to nasal cannula at any point, highlighting another key distinction from previous studies.

The b-CPAP pressure in the current program was set at 5–6 cmH₂O. While several trials, including ECLAT and Miami, reported higher CPAP success rates with increased pressures,^17,18^ the emphasis in this report was on optimizing the *effective pressure delivered to the infant’s lungs* rather than the set pressure alone. Effective pressure varies substantially by interface and technique. For example, an infant supported with short, curved, snugly fitted nasal prongs, a well-suctioned upper airway, and a chin strap in place is likely to receive higher and more consistent pressure compared with one using a narrow cannula with significant leak around the nostrils or partial upper airway obstruction from secretions. Previous studies have demonstrated that, when these precautions are applied, CPAP at 5–6 cmH₂O can provide adequate and reliable support.^19^

### Strengths and limitations

The study has several strengths, including contemporaneous comparisons between two NICUs with and without the use of b-CPAP, clear guidelines and training materials, and a gradual implementation strategy to enhance staff experience. The superior outcomes reported support the reliability of the b-CPAP program. We previously reported a sustained low incidence of BPD in premature infants with the use of a b-CPAP bundle.^4^ The present study is novel in that it not only confirms these findings but also outlines key elements for successful implementation, including structured simulation-based training, use of a bed-side checklist, and incorporation of a reliability audit system. Importantly, the study highlights the value of gradual adoption of the b-CPAP to allow teams to build experience. In addition, the involvement of two NICUs enabled a comparative analysis: one unit transitioning to b-CPAP and the other continuing with different CPAP, with outcomes assessed before and after b-CPAP introduction at both sites.

The definition of bronchopulmonary dysplasia (BPD) in this study followed the 2001 National Institute of Child Health and Human Development (NICHD) criteria, which define BPD based on the need for supplemental oxygen at 36 weeks’ postmenstrual age (PMA).^20^ Although newer definitions incorporating the level of respiratory support have been proposed, they were not applied in this study for several reasons. First, data collection and the statistical analysis plan were established before the introduction of these newer criteria. Second, the 2001 NICHD definition has been consistently used in major randomized clinical trials, allowing for direct comparison of effect sizes and contextualization of outcomes. For example, using this definition, the incidence of BPD was 40% in the SUPPORT trial among infants born at 24–27 weeks’ gestation and 29% in the COIN trial among those at 25–28 weeks, compared with only 6.9% in the present study.^21,22^ This consistent use of the NICHD definition facilitates benchmarking across studies and underscores the magnitude of improvement achieved following implementation. Third, concerns remain regarding the validity of newer definitions.^23^ A recent systematic review did not demonstrate superiority of the newer definitions over the 2001 NICHD criteria in predicting long-term outcomes.^24^ To address potential discrepancies arising from varying definitions, the investigators also reported data for infants who required noninvasive respiratory support without supplemental oxygen at 36 weeks’ PMA.

The study inherited some limitations, such as the lack of randomization, which is not feasible when training only half of the staff within each unit. Additionally, the study did not report long-term outcomes, and results should be interpreted with caution before generalization, as this was conducted within a single enterprise, though implementation at two sites suggests potential applicability elsewhere. The study was powered to detect outcome differences in the entire cohort before and after b-CPAP implementation and was not powered to assess changes in subgroup analyses. As a result, significant differences in subgroup analyses by gestational age and hospital site may have been missed. Although the number of transfers did not differ between the pre- and post-implementation periods, outcomes for these infants were not captured. While the regression model adjusted for multiple confounders, other factors were not accounted for, such as subtle changes in mechanical ventilation strategies or delivery room deaths. The incidence of sepsis, NEC, and PDA treatment did not differ between cohorts; however, unmeasured secular trends in neonatal outcomes over time may still have influenced the results.

## Conclusion

Survival without BPD improved in association with the implementation of a b-CPAP program that included training, bedside checklists, and reliability audits. The most significant improvement in BPD rates was observed in the youngest GA category (22–25 weeks). The gradual introduction of b-CPAP was crucial for developing staff experience. This approach should be considered in future trials of modalities that require significant training and experience.

## Supplementary information


Supplementary information


## Data Availability

The dataset generated during and/or analyzed during the current study is available from the corresponding author on reasonable request.
