# Supplementary material for: Structured implementation of a bubble-CPAP program to reduce bronchopulmonary dysplasia in preterm infants
Source: Pediatr Res. 2025 Dec 9;100(1):378–86. doi: 10.1038/s41390-025-04635-4 (PMC13524792; doi:10.1038/s41390-025-04635-4)
Supplement: Supplementary file 1 [file 41390_2025_4635_MOESM1_ESM.pdf]

Supplementary File

	Description	Page
Supplement A	Bubble continuous positive airway pressure (CPAP) guidelines	2-6
Supplement B	Daily Bedside Checklist	7
Supplement C	Table S1: Distribution of the study population by gestational age.	8

Supplement A

Bubble continuous positive airway pressure (CPAP) guidelines

Purpose

This clinical practice guideline is intended for care givers licensed independent practitioners (LIPs)/physicians utilizing bubble continuous positive airway pressure (CPAP) technology to treat respiratory conditions in neonates. It also defines the nursing role in the care of infants receiving b-CPAP.

Definitions

Licensed Independent Practitioner (LIP): A licensed provider acting within their scope of practice.

CPAP: A noninvasive system maintaining positive airway pressure during spontaneous breathing, typically 5–6 cm H_2O.

b-CPAP (Bubble CPAP): Achieved by submerging the expiratory limb in water, creating bubbling as humidified air passes through. Pressure corresponds to the depth of submersion.

Protocol Statement

- Application of b-CPAP requires a physician/licensed independent practitioner (LIP) order.
- Nursing staff are responsible for understanding the b-CPAP system.
- Nursing staff and respiratory therapists select the appropriate prong size and apply the prongs with a head cap.
- Early application of b-CPAP (at delivery) with pressures of at least 5 cm H_2O reduces the need for subsequent respiratory support.
- Infants on b-CPAP may be fed per physician/LIP order.
- Refer to "Set-Up for Infant on Bubble CPAP Using Nasal Prongs" SOP.

System Components: Heated circuit with inspiratory and expiratory limbs, binasal prongs, and head cap.

Cannulaide: Neonatal skin barrier and securement device sized by weight, ensuring seal and minimizing skin irritation.

Indications

Primary Indications

- Respiratory distress (tachypnea, flaring, grunting, retractions, cyanosis, atelectasis, increased oxygen requirement)
- Respiratory Distress Syndrome (surfactant deficiency disorder)
- Transient tachypnea of the newborn
- Apnea and bradycardia of prematurity
- Respiratory support post-extubation
- Partial or hemidiaphragm paralysis
- Tracheomalacia

Additional Indications

- Pulmonary edema
- Meconium aspiration syndrome
- Bronchopulmonary dysplasia

Contraindications

- Upper airway anomalies (e.g., cleft palate, choanal atresia, tracheoesophageal fistula, diaphragmatic hernia)
- Severe cardiac instability
- Scenarios where b-CPAP may fail in the delivery room (e.g., ≤ 24 weeks gestation, apnea due to maternal anesthesia)
- Respiratory failure

Guideline Statement

Target oxygen saturation for premature infants: 90–95%.

For infants with stage 3 retinopathy of prematurity (ROP), target oxygen saturation: 92–97%.

Bubble CPAP (BCPAP) Intervention Criteria

Indications for further intervention include:

- Increased work of breathing (e.g., tachypnea, severe retractions).
- $CO_2 \geq 65$ mmHg (by ABG or CBG).
- $FiO_2 \geq 60\%$ (or $\geq 50\%$ if the infant is < 48 hours old).

Before intervening, consider the following:

- Typically, more than one of the above indications should be present before initiating intervention.
- Is the infant < 4 hours old? Such infants may initially require higher oxygen concentrations, which typically decrease during the first few hours of life.
- Monitor the trend in oxygen requirement (increasing or decreasing). Allow the infant time to transition.
- For older infants, the primary indicator is an increasing oxygen requirement. Again, the trend is important.
- Evaluate the saturation profile: if >20% of saturations are below 90%, this suggests the need for intervention. Assess whether this trend is improving or worsening.

Weaning from Bubble CPAP

- Premature infants <30 weeks gestation and <1000 g birth weight are usually not weaned until they reach ~1300 g and a post-conceptual age of 32–33 weeks.
    - Initial CPAP wean: Infant on 21% O_2 for at least 48 hours and breathing comfortably.
    - Subsequent CPAP wean: Infant on 21% O_2 for at least 24 hours and breathing comfortably.
- Premature infants >1500 g birth weight may be weaned from CPAP once they tolerate 21% O_2 and are breathing comfortably.
- Initially remove the prongs and observe for increased work of breathing, tachypnea, or desaturation. If these signs occur, reconnect the prongs.
- A weaning trial may be repeated once daily. Do not use nasal cannula during the weaning process.
- Monitor the saturation profile trend closely during weaning.
- At 38 weeks gestation, a nasal cannula trial may be initiated in preparation for discharge.
- During oral feeds (PO) according to Infant Driven Feeding (IDF) scores, nasal cannula at 2 LPM can be used to support respiratory effort.

Protocol Implementation

Assessment

- Monitor respiratory status for increased work of breathing, elevated CO_2, and increased FiO_2.
- Confirm appropriate prong size.
- Check b-CPAP system each shift.
- Inspect skin at nares and septum during hands-on care.

Implementation

- Complete b-CPAP checklist at shift start and when caregiver changes (Attachment A). Keep at bedside.
- Elevate head of bed to 30°.
- Insert an 8 Fr orogastric tube at b-CPAP initiation; leave open for venting. Aspirate air before feeds.
- Provide developmental positioning and use a neck roll.
- Reposition the infant every 3–6 hours or during hands-on care.
- Declare "hands-off" periods if the infant is agitated.
- Gentle suctioning as needed, using the least invasive method first. Deep suction only if required, using an 8 Fr catheter. Use saline bullets and Ayr gel per physician/LIP order.
- Use stockinet chin strap or commercial equivalent.
- Provide oral care per NICU protocol.
- Change nasal prongs every 72 hours; respiratory therapist changes circuit/bubbler every 14 days.
- Introduce kangaroo care when stable on b-CPAP.

Cannulaide Application

- Select size based on weight.
- Change daily or as needed (~24-hour effectiveness).
- Clean and dry skin before application.
- Insert prongs through Cannulaide from non-adhesive side and place prongs in nares. Attach b-CPAP assembly.

Safety Checks

- Ensure b-CPAP remains correctly positioned.
- Prevent tubing from causing injury or burns.
- Maintain correct water levels and submersion depth.
- Monitor for Complications
- Nasal obstruction: suction as needed.
- Nasal septal erosion: confirm proper placement.
- Gastric distention: ensure orogastric tube patency.
- Pressure fluctuation: verify tubing placement.

Patient/Family Education

Provide education on b-CPAP therapy and distribute pamphlets when available.

Reportable Conditions

- Notify physician/LIP and RT of nasal septal breakdown.
- Notify physician/LIP and RT if respiratory distress worsens.
- Notify RT for circuit issues (e.g., absence of bubbling, incorrect CPAP level, or water accumulation in tubing).

Documentation (Electronic Health Record)

- Initiation and continuation of b-CPAP.
- Respiratory assessments.
- Positioning and skin integrity.
- Airway management.
- Reportable events.
- Family education.

Oversight and Responsibility

The Neonatology Division (Neonatal Executive Council) is responsible for the review, revision, updating, and operationalization of this guideline. The Nursing Institute is responsible for operationalizing nursing practices within the guideline.

References

- Aly H, Mohamed MA. Bubble continuous positive airway pressure. In: MacDonald MG, Ramasethu J, eds. Atlas of Procedures in Neonatology. 5th ed. Lippincott Williams & Wilkins; 2012.
- Bonner K, Mainous R. The nursing care of the infant receiving bubble CPAP therapy. Adv Neonatal Care. 2008;8:78–95.
- Wung J. Nuts and bolts of bubble CPAP [PowerPoint slides]. Columbia University Medical Center. Available at: http://healthcare-professionals.sw.org/resources/docs/divisionofeducation/events/neonatology-conference/2011/0900-nasal-CPAP-wung.pdf

Supplement B
Daily Bedside Checklist

Cleveland Clinic

Checklist is to be completed by the infant's nurse, each shift, while on CPAP

Current Weight:

DATE: ____________

SHIFT:			
❑ 1. Blended air/oxygen supply is appropriate			
❑ 2. Flowmeter is at 5-7 liters/min. **Document flow rate**			
❑ 3. Humidifier water level is correct ❑ Invasive ❑ Non-Invasive			
❑ 4. Excess rainout in the afferent/efferent tubing is drained			
❑ 5. Corrugated tubing is correctly placed			
❑ 6. Dial settings are clearly indicated.			
❑ 7. Sterile water level at mean fill line.			
❑ 8. Gas is bubbling continuously			
❑ 9. Head position is correct			
❑ 10. Neck roll is correct size and position.			
❑ 11. Hat fits snugly; no more than 1 finger's breadth space.			
❑ 12. Cannulaide is the right size and intact: ❑ change q 24 hrs ❑ adheres to the skin			
❑ 13. Moustache is right shape, size and intact:			
❑ velcro ❑ curved/thinned and ❑ not touching septum/nares			
❑ 14. Nasal prong size: If size not correct or prongs changed, note in comments section. *Patient should be measured using the size guide.* ❑ Correct size in use?			
❑ Document Babi Plus size	BP#	BP#	BP#
❑ Document Hudson prongs size	HP#	HP#	HP#
❑ 15. Nasal prongs are positioned correctly: not touching the septum or deviating to the side.			
❑ 16. Nasal septum changes (check all that apply) Mark abnormal findings →	❑ intact ❑ erythema ❑ pale ❑ erosion ❑ thin ❑ necrosis	❑ intact ❑ erythema ❑ pale ❑ erosion ❑ thin ❑ necrosis	❑ intact ❑ erythema ❑ pale ❑ erosion ❑ thin ❑ necrosis
❑ 17. Nare changes (check all that apply)	❑ intact ❑ thin ❑ pale ❑ erythema ❑ erosion ❑ necrosis ❑ obstructed L R	❑ intact ❑ thin ❑ pale ❑ erythema ❑ erosion ❑ necrosis ❑ obstructed L R	❑ intact ❑ thin ❑ pale ❑ erythema ❑ erosion ❑ necrosis ❑ obstructed L R

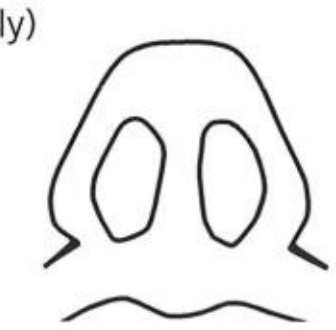

❑ 18. Provider and RT Notified of nare and/or septum changes. (Document name in comments)

❑ 19. Secretions
- ❑ clear to white, cloudy
- ❑ yellow to greenish
- ❑ bloody

Provider notified of secretion changes. (Document name in comments)

❑ 20. Date CPAP prongs are due for a change (due every 72 hrs)

❑ 21. Date CPAP setup due for change (due biweekly)

COMMENTS: Nurse Initials

____

____

Nurse Signature Initials

Size	Weight (grams)	French size
0	<700 g	10
1	700-1250 g	12
2	1250-2000 g	13
3	2000-3000 g	15
4	3000-3500 g	16
5	3500 g	20

Babi Plus Prongs Sizes

Size	Weight (grams)
0	<700
1	<700
2	700-1250
3	1250-2000
4	2000-3000
5	>3000
6	>3000
7	>3000

DAILY BEDSIDE CPAP CHECKLIST PWO 17112 2/18

Supplement C

Table S1: Distribution of the study population by gestational age.

Gestational Age	**22**	**23**	**24**	**25**	**26**	**27**	**28**	**29**	**30**	**31**	**32**	**33**	**34**	**Total**
Number	44	62	82	72	90	94	124	130	109	91	66	45	19	1028
Died <36wk	32 (73)	23 (37)	13 (16)	4 (6)	12 (13)	7 (7)	5 (4)	1 (1)	0 (0)	1 (1)	0 (0)	0 (0)	0 (0)	98 (9.5)
Transferred <36wk	4 (9)	8 (13)	19 (23)	14 (19)	7 (8)	16 (17)	12 (10)	13 (10)	13 (12)	10 (11)	8 (13)	1 (2)	0 (0)	125 (12)
Alive at 36wk	8 (20)	31 (50)	50 (61)	54 (75)	71 (79)	71 (76)	107 (86)	116 (89)	96 (88)	80 (88)	58 (88)	44 (98)	19 (100)	805 (78.5)
Home discharge	6 (14)	19 (31)	41 (50)	44 (61)	58 (64)	66 (70)	100 (81)	112 (86)	94 (86)	78 (86)	58 (88)	43 (96)	28 (97)	747 (72)
Died > 36wk	1 (2)	0 (0)	0 (0)	0 (0)	2 (2)	0 (0)	0 (0)	1 (1)	1 (1)	0 (0)	0 (0)	0 (0)	1 (3)	5 (.5)

Data are presented in numbers (%).
